# Examination of gender differences in patients with takotsubo syndrome according to left ventricular biopsy: two case reports

**DOI:** 10.1186/s13256-021-02856-9

**Published:** 2021-05-21

**Authors:** Tsutomu Murakami, Tomoyoshi Komiyama, Shingo Matsumoto, Hiroshi Kajiwara, Hiroyuki Kobayashi, Yuji Ikari

**Affiliations:** 1 Department of Cardiology, Tokai University School of Medicine, Isehara, Kanagawa 259-1193 Japan; 2 Department of Clinical Pharmacology, Tokai University School of Medicine, Isehara, Kanagawa 259-1193 Japan; 3 Department of Pathology, Tokai University School of Medicine, Isehara, Kanagawa 259-1193 Japan

**Keywords:** Takotsubo syndrome, Left ventricular biopsy, Gender differences, Microarray analysis, Gene expression, Cardiac injury, Pathology, H&E staining

## Abstract

**Background:**

Takotsubo syndrome is a stress-induced disease that makes up 2–3% of acute coronary syndrome cases. However, its onset mechanism remains unclear. Although females are overwhelmingly affected, males end up having more cardiac complications.

**Case presentation:**

We examined the differences in stress responses in the myocardium between sexes in patients with takotsubo syndrome. We biopsied samples from an over 70-year-old Japanese male and an over 80-year-old Japanese female. Tissues from the left ventricle apex in the acute phase and the apical ballooning-type were examined using histopathology and deoxyribonucleic acid (DNA) microarray analysis. Our data showed that left ventricular ejection fractions were 38% and 56%, and peak creatinine kinase concentrations during hospitalization were 629 U/L and 361 U/L, for the male and female patient, respectively. The pulmonary capillary wedge pressure was 26 mmHg and 11 mmHg for the male and female patient, respectively. Negative T did not return to normal in the male subject after 6 months. Histopathology results indicated that contraction band necrosis and lymphocyte infiltration were more common in the male subject.

**Conclusions:**

We noticed that possible differences may exist between male and female patients using pathological examination and some DNA analyses. In particular, it may help treat acute severity in males. We will elucidate the mechanism of takotsubo syndrome development by increasing the number of samples to support the reliability of the data in the future.

**Supplementary Information:**

The online version contains supplementary material available at https://doi.org/10.1186/s13256-021-02856-9.

## Background

There are still many unclear issues about the onset mechanism of takotsubo syndrome (TTS), and a novel approach is desired because there are no specific treatments or preventions. Therefore, we wanted to clarify the pathological condition by comparing the similarities and differences between male and female patients with TTS, focusing on why the disease is overwhelmingly common in females and why males end up having a more severe condition.

**Fig. 1.**
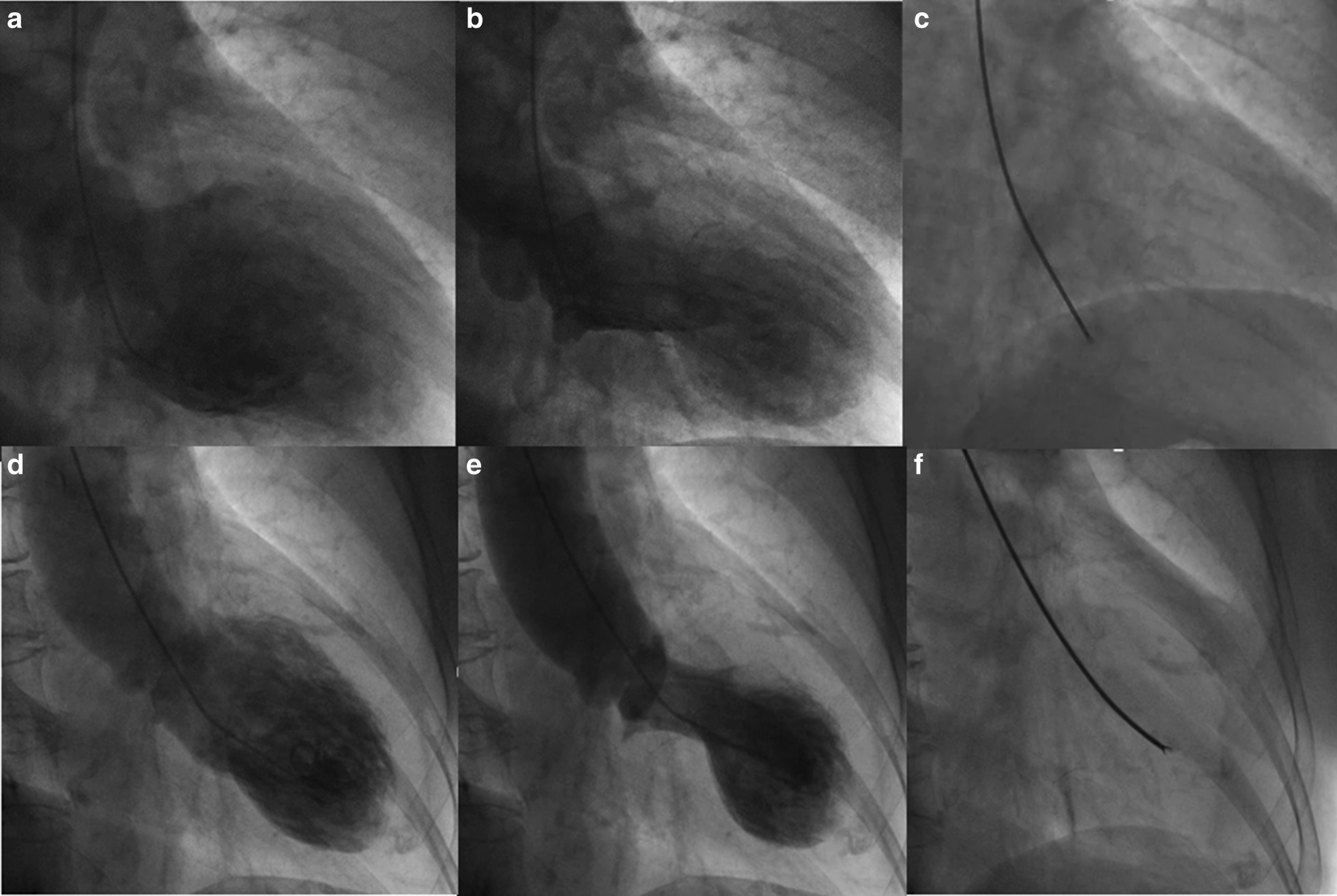
Left ventricle angiography and catheter biopsy. The diastolic phase of the left ventricle (**a** and **d**), the systolic phase of the left ventricle, similar to takotsubo (**b** and **e**), and biopsy from the lesion of apical ballooning (**c** and **f**) are shown. Male: **a**, **b**, and **c**, female: **d**, **e**, and **f**

Takotsubo cardiomyopathy (TC), which results from stress, was first announced to the world by a Japanese doctor in 1990 [1, 2]. It was named takotsubo because it is shaped like a takotsubo (octopus pot), a traditional trap for catching octopus in Japan [3, 4]. The syndrome causes apical wall motion, which prevents contraction during systole, and the base of the heart becomes hypercontracted (Fig. 1).

The disease is now known worldwide as TC or TTS [5–8]. The wall motion naturally improves in about 2 weeks after the onset, and hence the prognosis is good; therefore, there is no progress in studying the pathophysiology [9, 10]. However, we think that heart failure, cardiogenic shock, and arrhythmia may lead to death in the acute phase. The percentage of heart-related deaths during hospitalization is 3.7%, and about 35% of TC are associated with heart failure [11]. TTS is characterized by a reversible left ventricular dysfunction, predominant in females with a 76–90% occurrence [5, 12, 13]. There are also some cases from recent reports that do not show a good prognosis [14, 15]. In addition, we previously reported that TTS cases in males are more severe than those in females [13]. This is considered to be due to the difference in stress responses between males and females. Furthermore, as TTS is caused by stress, it is considered that estrogen decline is also among the causes because of the involvement of excess secretion of catecholamines and the high proportion of cases in postmenopausal females [5, 8, 16]. The prevalence of TTS is approximately 2–3% in all patients presenting with suspected acute coronary syndrome [17]. Therefore, male patients with TTS are rare, and their clinical features remain to be determined. It was recently reported that cardiac complications, such as cardiovascular death, severe pump failure, and ventricular arrhythmia, are more common in male patients than in female patients with TTS [13]. TTS occurs in older postmenopausal women, so decreased estrogen levels are thought to contribute to TTS onset, and the theory remains promising [18, 19]. However, the hypoestrogenic hypothesis cannot explain the onset mechanism of male TTS. In addition, we previously reported that males have a higher rate of TTS associated with heart failure, arrhythmia, and in-hospital mortality than females [13]. This is believed to be due to a mechanism that increases the severity of male TTS. Even now, a quarter of a century later since the discovery of TTS, the detailed pathophysiology has not been elucidated, and there is currently no cure or prevention. An unprecedented approach from a new perspective is necessary.

Herein, by examining the gender differences, we aimed to clarify the similarities and differences from various angles, leading to the elucidation of the disease onset mechanism. Therefore, to achieve clarification, we attempted to observe changes in the left ventricle myocardium during the acute phase via left ventricle biopsy. We performed myocardium biopsy from the apical of the left ventricle during the acute phase in apical ballooning-type TTS (Fig. 1) and investigated the pathology using hematoxylin–eosin (H&E) staining and deoxyribonucleic acid (DNA) microarray. In addition, we obtained clinical data, including the clinical background, laboratory, echocardiography, and catheter data, and surveyed the gender differences. The findings of this case report will be very useful for the treatment of TTS in the acute phase and contribute to novel technologies currently being studied.

## Case presentation

### Case description, timeline, and clinical intervention

We studied two patients with TTS; one patient was a male > 70 years of age, and the other was a female > 80 years of age (Table 1). This case report was conducted according to the guidelines of the Declaration of Helsinki, and approved by the Institutional Review Board (or Ethics Committee) of Tokai University, Japan (protocol code 15R135 and date of approval). Informed consent was obtained from both the subjects.

**Table 1:** Clinical physiology of the male and female subjects

	Male	Female
Age (years)	> 70	> 80
Preceding stress	None	Fall
*Catheter data*		
Catheter day	2017.6	2016.2
Coronary artery disease	None	None
Aorta (s/d/m), mmHg	141/86/102	127/71/96
Left ventricular pressure (s/d/m), mmHg	141/5/25	129/1/20
PCWP (s/d/m), mmHg	29/35/26	14/12/11
Pulmonary artery pressure (s/d/m), mmHg	39/26/32	33/14/21
Right ventricular pressure (s/d/m), mmHg	48/9/12	36/3/17
Right atrium pressure (s/d/m), mmHg	13/12/11	8/7/6
Cardiac index, L/min/m^2^	1.85	1.79
*Laboratory data*		
Max CK during hospitalization, U/L	629	361
Max CK-MB during hospitalization, U/L	44	33
White blood cell count, /µL	14,200	12,800
Brain natriuretic peptide, pg/mL	52.6	38.7
*Transthoracic echocardiography*		
Ejection fraction, %	38	56
Left ventricular diastolic diameter, mm	68	41
Left ventricular systolic diameter, mm	42	22

*PCWP*, pulmonary capillary wedge pressure; *s/d/m*, systolic/diastolic/mean; *CK*, creatine kinase

The main complaint of the male patient was chest pain, and that of the female patient was disturbance of consciousness. While the female patient demonstrated preceding physical stress, it was not observed in the male subject. The male patient had a medical history of hypertension and hyperuricemia, and the female had a medical history of hypertension and dyslipidemia. Family and psychosocial history was not obtained for both patients. The male patient was a smoker, whereas the female patient had never smoked. Both patients drink occasionally. Physical examination revealed no significant findings such as heart murmur or edema in either patient. The end expiratory wheeze was auscultated in the male patient. An electrocardiogram (ECG) of the male patient was taken at the time of the visit (on attack), and the next ECG was taken 2 days after the onset in the morning. The next ECG was taken 2 months after the onset, and once more 6 months after the onset. The last ECG was performed 3 years after the initial attack. The timing of ECG for the female patient was the same as that for the male patient; in addition, an ECG was taken 1 month after the onset. Mild congestion was noted in the male patient, and chest X ray of the female patient revealed no signs of congestion or pleural effusion. Left ventricular biopsy was performed after coronary angiography and left ventriculography via the right radial artery. After inserting 5-Fr × 98 cm sheathless catheter introducer (Medikit Corporation, Japan) in the left ventricle via the right radial artery, the biopsies were conducted using 6-Fr × 105 cm biopsy forceps (Cordis Corporation, USA). The time of the biopsy was 11:00 a.m. for the female subject and 12:00 p.m. for the male subject. The diagnosis of TTS was performed on the basis of characteristic changes of the ECG and left ventricular angiography. Moreover, there was no TTS history in the family.

Vital signs, laboratory data, transthoracic echocardiography (TTE) data, and Swan–Ganz catheter information are presented in Table 1. White blood cell (WBC) count, peak creatinine kinase (CK), and brain natriuretic peptide (BNP) levels were higher in the male patient. In the male patient, the ejection fraction of the left ventricle was lower, and the left ventricle was larger compared with those of the female patient. Moreover, the mean pulmonary capillary wedge pressure (PCWP) was higher in the male patient (26 mmHg) than in the female patient (11 mmHg), revealing heart failure. The left ventricular ejection fraction was 38% for the male patient and 56% for the female patient, and the peak CK during hospitalization was 629 U/L and 361 U/L for the male and female patients, respectively (Table 1).

**Fig. 2:**
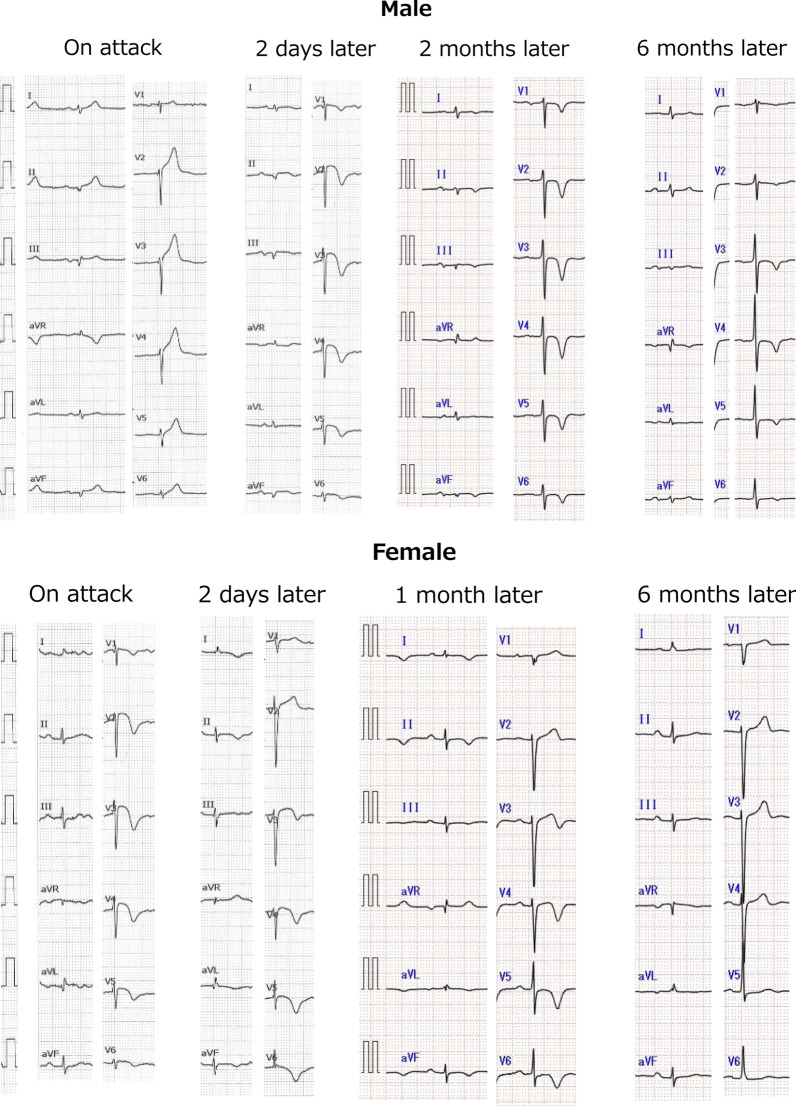
Follow-up comparison within 6 months using electrocardiography. Upper: male, lower: female

Table 2 and Fig. 2 show an ECG follow-up of the two patients 6 months after admission. In the follow-up of the male subject and the female subject, results of ECG examinations were similar after 2 days, 1 month, and 2 months. However, the negative T of the male subject did not return to normal after 6 months. For the male patient, negative *T* was not found after 3 years (May, 2020).

**Table 2:** Follow-up comparison of the male and female subjects using electrocardiogram

	Male	Female
On attack	1. ST elevations in V2-6, II, III, and aVF were observed	1. ST elevations in V2-6 were observed 2. Negative T in V1-6 were observed
2 days later	1. ST elevations in V2-6 were decreased, and negative T in V1-6 were observed	1. ST elevations in V2-6 were decreased 2. Negative T in V3-6, I, II, and aVL were observed
Male: 2 months later Female: 1 month later	1. Negative T in V1-6, II, III, and aVF were observed	1. Negative T in V3-6 I, II, aVL, and aVF were observed
6 months later	1. Negative T in V1-6 were observed 2. Negative T not yet returned to normal	1. ECG returned to almost normal

### Severity of cardiac injury from pathology analysis via H&E staining

**Fig. 3:**
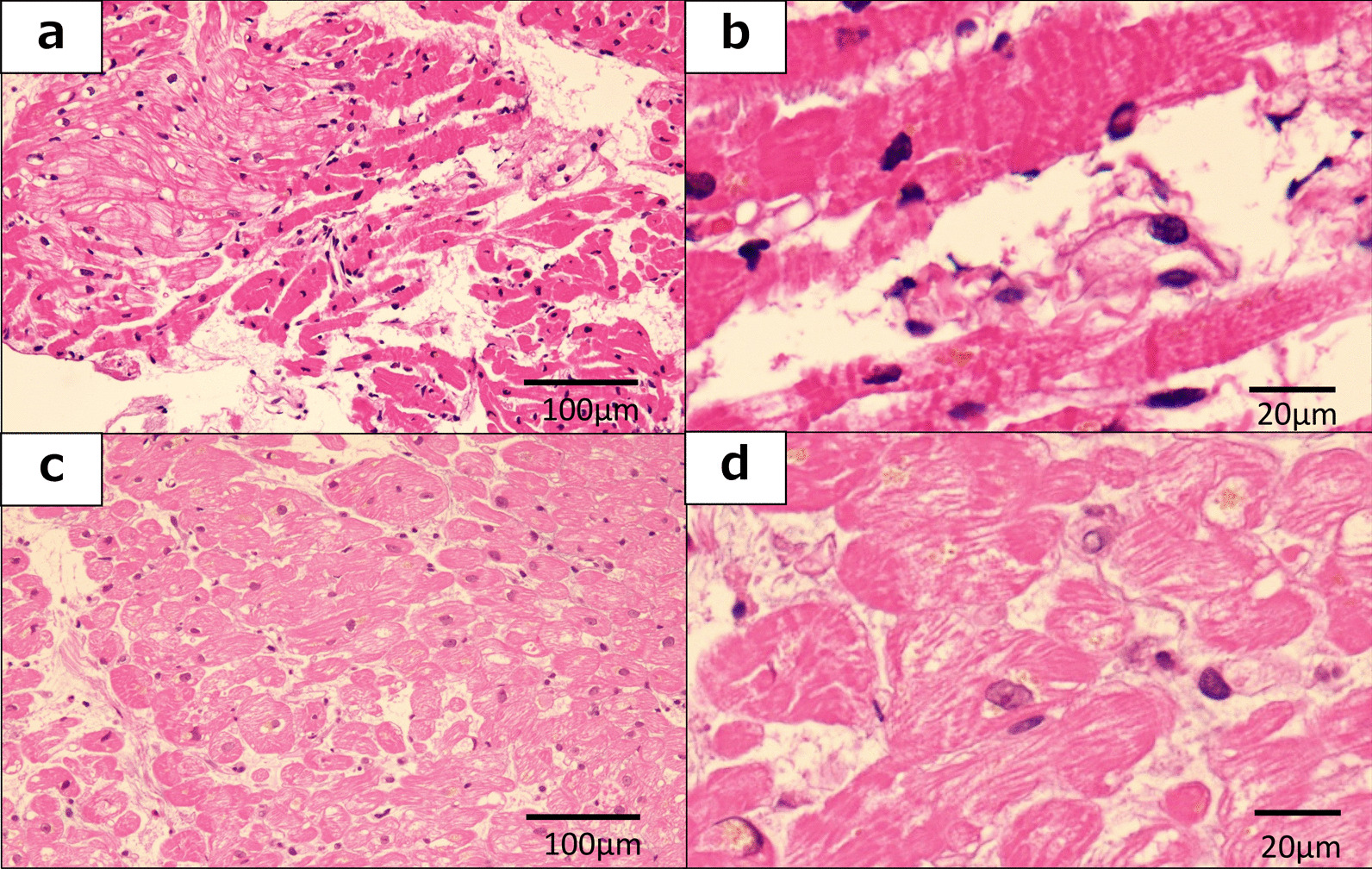
Pathological examination using hematoxylin–eosin (H&E) staining. Photo A reveals microscopic findings in the myocardium of the male patient (**a**: original magnification 400×, **b**: original magnification 1000×). A necrotic lesion (darker pink area) was found in the male patient, which was accompanied by slight lymphocytic infiltration (**a** and **b**). Contraction band necrosis was also found at high-power view (**b**). Photos **c** and **d** reveal microscopic findings in the myocardium of the female patient (**c**: original magnification 400×, **d**: original magnification 1000×). In the female patient, the necrotic lesion was very limited and was not accompanied by lymphocytic infiltration (**c**). Necrotic lesion revealed an acidophilic change in the myometrium. However, contraction band necrosis was not apparent at high-power view (**d**)

The biopsied specimens were fixed in 10% neutral formalin solution overnight, dehydrated in ethanol series, and cut into 6-μm-thick paraffin blocks using a microtome. Sections were dehydrated in distilled water and stained with H&E. The pathological findings via H&E staining are shown in Fig. 3. In the male patient with TTS, the necrosis was broader and was accompanied by lymphocytic infiltration (Fig. 3a), and contraction band necrosis was found at a high-power view (Fig. 3b). In contrast, in the female patient, a few necrotic portions without lymphocytic infiltration were observed (Fig. 3c, d).

### Microarray analysis and gene expression analysis based on DAVID

Total ribonucleic acid (RNA) was extracted from heart tissues using TRIzol (Invitrogen, Carlsbad, CA, USA), followed by Qiagen RNeasy (Qiagen, Valencia, CA, USA). Each RNA preparation was tested for degradation using the Agilent 2100 Bioanalyzer (Agilent Technologies, Palo Alto, CA, USA). Total RNA (50 ng) was amplified using the One-Color Low Input Quick Amp Labeling Kit (Agilent Technologies) and purified using the RNeasy Mini Kit (Qiagen) (Additional file 1: Table S1). Preparation of probes and hybridization was performed following the One-Color Microarray Based Gene Expression Analysis Manual Ver. 6.5 (Agilent Technologies). We used the Sure Print G3 Agilent 8x60K Human Microarrays (G4852A-028004). Images were captured using an Agilent Microarray Scanner, and spots were quantified using Feature Extraction Software (Agilent Technologies) (Additional file 2: Table S2).

For enrichment analysis, we created lists annotated to *Homo sapiens* genes. Listed genes were submitted to Database for Annotation, Visualization and Integrated Discovery (DAVID) functional annotation database (http://david.abcc.ncifcrf.gov/), providing a broad, unguided test against primarily gene ontology (GO) groups and Kyoto Encyclopedia of Genes and Genomes (KEGG) pathways [20]. Extraction of GO gene groups and KEGG pathways caused the expression levels to increase fourfold. The results were only considered statistically significant when the *P*-value was < 0.05. Data were analyzed using independent samples *t*-test with *P* < 0.05 considered significant. Error bars indicate the standard error of the average anthocyanin contents (Additional file 1: Table S3–S6).

For the female patient, from the GO analysis, we confirmed significant fluctuations in the gene groups related to the cell membrane, such as the extracellular region (*P*-value = 1.32 × 10^−18^), extracellular space (*P*-value = 2.52 × 10^−15^), collagen trimer (*P*-value = 2.15 × 10^−10^), extracellular exosome (*P*-value = 1.03 × 10^−04^), plasma membrane (*P*-value = 0.002588), and an integral component of plasma membrane (*P*-value = 0.00353), the expression of which increased more than fourfold (Additional file 1: Table S3).

**Fig. 4.**
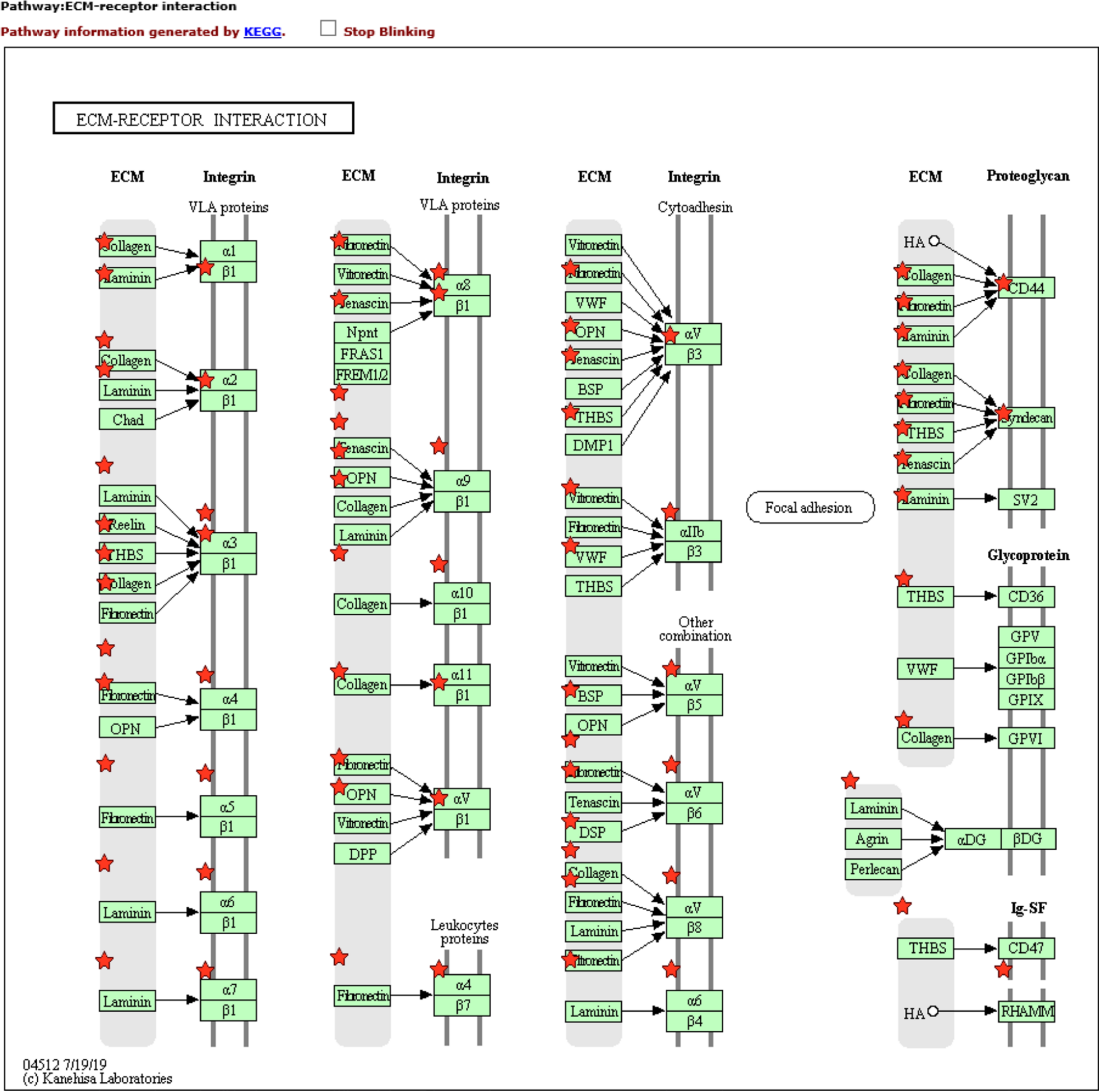
Extracellular matrix-receptor interaction in the female subject obtained via Kyoto Encyclopedia of Genes and Genomes (KEGG) analysis. *ECM*, extracellular matrix [20]. The image of map04512 ECM-receptor interaction is reproduced with permission from KEGG

From KEGG analysis results for the female patient, we confirmed the extracellular matrix (ECM)-receptor interaction (*P*-value = 1.23 × 10^−07^) pathway; cell–cell interaction pathways, such as cell adhesion molecules (CAMs) (*P*-value = 8.61 × 10^−05^), cytokine–cytokine receptor interaction (*P*-value = 0.004895), focal adhesion, and protein digestion (*P*-value = 0.004979); and absorption pathways (*P*-value = 0.005905) (Additional file 1: Table S4). In particular, in the ECM–receptor interactions, the interaction between the ECM and integrins was strong, as seen in Fig. 4. Moreover, it influenced the interaction of collagen, laminin, and integrin receptors.

For the male patient, from the GO analysis, we found that expression-variable genes (the expression of which increased fourfold) related to the integral component of the membrane (*P*-value = 4.46 × 10^−05^), the plasma membrane (*P*-value = 2.26 × 10^−04^), the extracellular space (*P*-value = 0.001221), and the integral component of the plasma membrane (*P*-value = 0.003399) showed changes in expression. In addition, a change was confirmed in the mitochondrion gene group related to ATP synthesis (Additional file 1: Table S5).

KEGG pathway analysis of the male patient showed that metabolic pathways (*P*-value = 0.005675); valine, leucine, and isoleucine degradation (*P*-value = 0.006051); beta-alanine, arginine, and proline metabolism (*P*-value = 0.00992); and cardiac muscle contraction (*P*-value = 0.011573) pathways were associated with metabolic pathway changes (Additional file 1: Table S6). In particular, the change in the expression level of metabolic pathways was more substantial.

## Discussion

We conducted a study considering the difference in stress responses in the myocardium of patients with TTS, which is caused by gender differences. We investigated the gene expression levels in the myocardium to determine gender differences and elucidate the pathophysiology.

The severity of cardiac injury was evaluated by monitoring the peak CK concentration during hospitalization due to acute coronary syndrome. TTS is considered an acute coronary syndrome; therefore, peak CK is an indicator of the severity of the cardiac injury. In addition, we previously reported that high WBC and BNP levels are associated with poor clinical outcomes in patients with TTS [11]. In the current cases, peak CK, WBC, and BNP levels were higher in the male subject; therefore, the cardiac injury was more severe in the male subject. Left ventricular ejection fractions were 38% and 56% for the male and female subject, respectively, and the peak CK during hospitalization was 629 U/L and 361 U/L for the male and female subject, respectively. The right heart catheter during the acute phase showed that the PCWP was 26 mmHg for the male patient and 11 mmHg for the female patient, suggesting heart failure in the male subject.

Pathological examination by H&E staining showed contraction band necrosis more frequently in the male patient, which suggested cardiac injury, and we observed increased lymphocyte levels instead of eosinophil levels. In the female patient, contraction band necrosis was not observed. It was previously reported via autopsy and endomyocardial biopsy that the myocardial lesions of TTS are characterized mainly by individual myocytes or a group of myocytes with increased eosinophil staining, contraction band formation, necrosis, and rupture [21]. The tissues for pathological examination are mostly obtained from autopsy cases [21, 22]. It was also previously reported that the tissues are taken by endomyocardial biopsy at the acute phase if any of the biopsy lesions are in the right ventricle despite left ventricle dysfunction [9, 23]. Therefore, to our knowledge, this is the first report of biopsy of the left ventricle in apical ballooning-type TTS in the acute phase. We performed a left ventricle apical biopsy, which demonstrated akinetic lesions in the apical ballooning-type of TTS during the acute phase.

Then, we also performed DNA microarray analysis of the left ventricle tissue of the male and female subjects. In the GO analysis, we confirmed that the plasma membrane and integral components of the plasma membrane were common between the male and female patients. We considered that this was strongly damaged at the onset of TTS. Moreover, in the male patient, hemoglobin complex, extracellular space, and mitochondrion genes were confirmed by GO.

A change was confirmed in the mitochondrion gene group related to ATP synthesis. In the male patient, metabolic pathways were found to be more elevated than in the female patient, as revealed by KEGG pathway analysis. In the GO analysis, it was confirmed that expression-variable genes related to the integral components of the membrane were higher in the male subject than in the female subject.

In contrast, ECM–receptor interaction increased more substantially in the female subject than in the male subject, as revealed by the KEGG analysis. Differences in the expression of collagen, proteoglycan, and laminin in the constituent molecules of the basement membrane were confirmed. ECM improves the function of cardiomyocytes by adding laminin, as reported before. In addition, the affecting collagen functions were confirmed by GO analysis (Additional file 2: Table S2). Furthermore, changes in the expression levels of various integrin subunits that play an important role in cell adhesion were confirmed. This may affect intracellular or extracellular signals. It is also conceivable that the expression levels may have changed over time since the onset of TTS.

Did the changes in the gene expression cause TTS? Or did the gene expression change because TTS developed? These results indicate that takotsubo syndrome in males affects mitochondria, which are involved in energy production, regulation of intracellular calcium ion concentration, lipid oxidation, and immune response. In the female subject, we found that it affected the integrins, collagen, proteoglycans, and laminin that make up the basement membrane. Furthermore, GO analysis confirmed that the differentially expressed genes related to the plasma membrane were more elevated in the female subject than in the male subject. TTS prognosis was worse during hospitalization in the male than in the female subject, probably because metabolic pathways and plasma membranes were affected in the male subject. It is interesting to note that mitochondria were also affected.

Mitochondrial DNA is known to be involved in aerobic respiration and ATP synthesis. It also plays an important role in apoptosis. Part of mitochondrial DNA and its gene products are also localized on the cell surface, and a mutation is specifically excluded by the innate immune system [9]. In humans, hundreds and thousands of mitochondria are present in cells actively involved in metabolism, such as those of the liver, kidney, muscle, and brain [24–27]. Inside the mitochondria, surrounded by the inner membrane, is a matrix, and there are many processes involved in mitochondrial metabolic functions, such as the Krebs cycle and β-oxidation [28–30]. The citric acid cycle is a cyclic metabolic pathway consisting of nine steps performed in the mitochondrial matrix [31–34]. The citric acid cycle is the most important biochemical cycle for aerobic metabolism, found in all organisms that metabolize oxygen. Of note, TTS is possibly a result of the abnormal expression of enzymes related to mitochondrial structure and metabolic function.

Based on the above, the male patient with TTS exhibited changes in the expression levels of enzymes related to mitochondrial metabolic function. This might indicate a poor prognosis during hospitalization.

Based on pathological examination and DNA microarray analysis, it is possible that cardiac complications are more common in male patients with TTS. On pathological examination, cardiac band necrosis and infiltration of lymphocytes were more common in the male patient than in the female patient. We will continue to search for patients with takotsubo and accumulate data to clarify differences in the onset mechanism between males and females. Moreover, we believe that male and female hormones are involved in the onset of takotsubo, so we would like to consider this as well.

## Conclusions

It is important to note that the sample used was from the apex tissue of the left ventricle in the acute phase of TTS. Using pathological examination and DNA analyses, we observed possible differences between the male and female patients. The results of our research contribute to new developments in future studies and may help treat acute severity in males. We will elucidate the mechanism of TTS development by increasing the number of samples to support the reliability of the data in the future.

## Supplementary Information

**Additional file 1: Table S1.** Results of the RNA sample quality test, Table S3: The results of gene ontology analysis in the female subject, Table S4: The results of Kyoto Encyclopedia of Genes and Genomes analysis in the female subject, Table S5: The results of the male subject via gene ontology analysis, and Table S6: The results of Kyoto Encyclopedia of Genes and Genomes analysis in the male subject (Word, .doc)

**Additional file 2: Table S2.** Gene expression analysis: list of genes.

## Data Availability

Not applicable.

## Abbreviations

BNP: Brain natriuretic peptide
CAMs: Cell adhesion molecules
CK: Creatinine kinase
ECG: Electrocardiogram
ECM: Extracellular matrix
GO: Gene ontology
H&E: Hematoxylin–eosin
KEGG: Kyoto Encyclopedia of Genes and Genomes
PCWP: Pulmonary capillary wedge pressure
TC: Takotsubo cardiomyopathy
TTE: Transthoracic echocardiography
TTS: Takotsubo syndrome
WBC: White blood cell
