# Supplementary material for: Examination of gender differences in patients with takotsubo syndrome according to left ventricular biopsy: twocase reports
Source: J Med Case Rep. 2021 May 21;15:281. doi: 10.1186/s13256-021-02856-9 (PMC8139097; doi:10.1186/s13256-021-02856-9)
Supplement: Supplementary file 1 — Additional file 1: Table S1. Results of the RNA sample quality test, Table S3: The results of gene ontology analysis in the female subject, Table S4: The results of Kyoto Encyclopedia of Genes and Genomes analysis in the female subject, Table S5: The results of the male subject via gene ontology analysis, and Table S6: The results of Kyoto Encyclopedia of Genes and Genomes analysis in the male subject (Word, .doc) [file 13256_2021_2856_MOESM1_ESM.docx]

Additional Material

# Additional methods

**Gene expression via DNA microarray**

**Gene Array Hybridization**

**RNA absorbance measurement**

Absorbance was measured in RNA mode using NanoDrop 1000.

**RNA quality inspection**

RNA quality inspection was performed using Agilent 2100 BioAnalyzer series II (Agilent Technologies, Santa Clara, CA, USA):

1. One microliter of dye was added to the dispensed gel and centrifuged at 13,000 × *g* for 10 min.

2. The RNA sample was dispensed into a sterilized tube.

3. The collected RNA sample was incubated at 70 °C for 3 min, then rapidly cooled on ice for 3 min and heat-treated.

4. A chip preparation stand was prepared, and the Lab Chip was set.

5. Gel-Dye Mix was added onto the chip.

6. A 5-μL marker (internal standard) was dispensed into the ladder well, and all sample wells.

7. RNA samples were dispensed into each sample well.

8. One microliter of the ladder was dispensed into ladder wells.

9. LabChip was agitated for 1 min using a LabChip mixer.

10. Immediately after preparation, the chip was placed on a BioAnalyzer and measured.

**cDNA synthesis, cRNA labeling, and amplification**

cDNA was synthesized using Low Input Quick Amp Labeling Kit (Agilent Technologies), labeled, and then amplified.

1. Total RNA, T7 Promoter Primer, and Nuclease Free Water were added as shown below.

Total RNA requirement (Table S1)

T7 Promoter Primer (1.8 μL)

Nuclease Free Water (up to 3.3 μL)

2. Two microliters of RNA Spike (for 1 color) was added to the sample.

3. After 10 min of incubation at 65 °C, it was heat-denatured by placing on ice for 5 min.

4. During this time, the next cDNA master mix was prepared.

Required amount of reaction:

5× First-Strand Buffer 2.0 μL

0.1 M DTT 1.0 μL

10 mM dNTP mix 0.5 μL

AffinityScript 1.2 μL

Total of 4.7 μL

5. cDNA master mix of 4.7 μL was added to the heat-denatured RNA and incubated in a 40 °C water bath for 2 h.

6. The tube was removed from the water bath and incubated at 70 °C for 15 min and cooled on ice for 5 min.

7. During this time, the following transcription master mix was prepared.

Required amount of reaction:

Nuclease Free Water 0.75 μL

5× Transcription Buffer 3.2 μL

0.1 M DTT 0.6 μL

NTP Mix 1.0 μL

T7 RNA polymerase Mix 0.21 μL

Cyanine 3 CTP 0.24 μL

Total 6.0 μL

8. Six microliters of transcription mix was added to each reaction tube.

9. The sample was incubated for 2 h in a 40 °C water bath, protected from light.

**Purification of labeled cRNA**

Labeled cRNA was purified using RNeasy mini spin columns, QIAGEN.

1. Nuclease Free water of 84 μL was added to the reaction tube.

2. RLT Buffer of 350 μL was added to the reaction tube.

3. Ethanol (96–100% purity) of 250 μL was added to the reaction tube.

4. cRNA sample of 700 μL was transferred to the column and centrifuged at 13,000 rpm for 1 min.

5. The flow-through was transferred to another tube, and the column was returned to the collection tube.

6. RPE Buffer of 500 μL was added and centrifuged at 13,000 rpm for 1 min.

7. Steps 5 and 6 were repeated.

8. The column was transferred to a 1.5 mL tube.

9. of Nuclease-free water of 30 μL was added and allowed to stand for 1 min.

10. The sample was centrifuged for 1 min at 13,000 rpm to elute.

**Labeled cRNA quality inspection**

**1) cRNA quality inspection**

RNA quality testing was performed using an Agilent 2100 BioAnalyzer series II.

1. One microliter of dye was added to the dispensed gel and centrifuged at 13,000 × *g* for 10 min.

2. A chip preparation stand was prepared, and the Lab Chip was set.

3. Gel-Dye Mix was filled into the chip.

4. A 5-μL of marker (internal standard) was dispensed into the ladder well, and all sample wells.

5. RNA samples were dispensed into each sample well.

6. One microliter of the ladder was dispensed into the ladder well.

7. LabChip was agitated for 1 min using LabChip mixer.

8. Immediately prepared chip was set in BioAnalyzer and measured.

**2) cRNA absorbance measurement**

Using NanoDrop 1000, the absorbance was measured in Microarray Measurement mode, and the cRNA sample was stored at −80 °C.

**Hybridization**

Hybridization was performed using Gene Expression Hybridization Kit, Agilent Technologies.

1. 2 × cRNA target solution was prepared as follows:

per tube

Cyanine3-labeled cRNA 600 ng

10× Blocking Agent 5 μL

Nuclease Free Water up to 24 μL

2. One microliter of 25× fragmentation buffer was added to the 2× cRNA target solution.

3. Incubated for 30 min in a 60 °C water bath.

4. 2× hybridization buffer of 25 μL was added to stop fragmentation.

5. Then, 40 μL was applied to the Microarray, and hybridization was performed stirring in a hybridization oven at 65 °C for approximately 17 h.

**Slide glass cleaning**

Gene Expression Wash Buffer (Agilent Technologies) was used to wash glass slides.

Slides were washed with Agilent Gene Expression Wash Buffer 1 at room temperature (approximately 26 °C) for 1 min.

They were washed with Agilent Gene Expression Wash Buffer 2 at 37 °C for 1 min.

The slide was pulled out and air-dried.

**Scanning**

Scanning was performed with Agilent Technologies Microarray Scanner with Scan Resolution: 3 μm, TIFF file dynamic range: 20 bit.

**Digitization**

Each spot was quantified using Agilent Feature Extraction 12.0.3.1.

**Normalize (75Percentile Shift)**

Raw data output was digitized using the Agilent Feature Extraction (12.0.3.1) software.

The text file was imported into GeneSpring and normalized with the 75^th^ percentile shift.

**Creation of individual sample data**

1. The value of 75Percentile Shift output from GeneSpring adjusted by log2 was converted to a true number in Excel.

2. Seven types (gIsSaturated, gIsFeatNonUnifOL, gIsBGNonUnifOL, gIsFeatPopnOL, gIsBGPopnOL, gIsPosAndSignif, gIsWellAboveBG) of flags were selected from each text file of raw data and pasted into Excel.

3. These flags were color-coded for each sample.

# Additional Tables

Table S1. Results of the RNA sample quality test

| No. | Sample name | Nanodrop | | | Bioanalyzer | | |
| --- | --- | --- | --- | --- | --- | --- | --- |
|  |  | A260/A280 | A260/A230 | ng/μL | 28S/18S | RIN | ng/μL |
| 1 | T1 | 1.74 | 2.14 | 149.5 | 1.0 | 7.6 | 95.4 |
| 2 | T2 | 1.59 | 2.14 | 99.2 | 0.7 | 7.7 | 50.4 |

Table S3: The results of gene ontology analysis in the female subject

| **Category** | **Term** | **Count** | **%** | **P-Value** | **Bonferroni** | **Benjamini** |
| --- | --- | --- | --- | --- | --- | --- |
| GOTERM_CC_DIRECT | GO:0005576~extracellular region | 135 | 15.93861 | 1.32E-18 | 5.28E-16 | 5.28E-16 |
| GOTERM_CC_DIRECT | GO:0005615~extracellular space | 113 | 13.3412 | 2.52E-15 | 1.02E-12 | 5.11E-13 |
| GOTERM_CC_DIRECT | GO:0005578~proteinaceous extracellular matrix | 38 | 4.486423 | 1.26E-11 | 5.03E-09 | 1.68E-09 |
| GOTERM_CC_DIRECT | GO:0005581~collagen trimer | 21 | 2.479339 | 2.15E-10 | 8.60E-08 | 2.15E-08 |
| GOTERM_CC_DIRECT | GO:0031012~extracellular matrix | 38 | 4.486423 | 2.36E-10 | 9.42E-08 | 1.88E-08 |
| GOTERM_CC_DIRECT | GO:0005788~endoplasmic reticulum lumen | 21 | 2.479339 | 4.89E-05 | 0.019383 | 0.003257 |
| GOTERM_CC_DIRECT | GO:0000775~chromosome, centromeric region | 11 | 1.298701 | 5.33E-05 | 0.02108 | 0.003039 |
| GOTERM_CC_DIRECT | GO:0009897~external side of plasma membrane | 22 | 2.597403 | 7.16E-05 | 0.028249 | 0.003576 |
| GOTERM_CC_DIRECT | GO:0070062~extracellular exosome | 145 | 17.11924 | 1.03E-04 | 0.040285 | 0.004558 |
| GOTERM_CC_DIRECT | GO:0031093~platelet alpha granule lumen | 10 | 1.180638 | 2.18E-04 | 0.083684 | 0.008701 |
| GOTERM_CC_DIRECT | GO:0005604~basement membrane | 11 | 1.298701 | 8.36E-04 | 0.284254 | 0.029945 |
| GOTERM_CC_DIRECT | GO:0000777~condensed chromosome kinetochore | 11 | 1.298701 | 0.001767 | 0.507041 | 0.05724 |
| GOTERM_CC_DIRECT | GO:0072562~blood microparticle | 15 | 1.770956 | 0.002114 | 0.571079 | 0.063039 |
| GOTERM_CC_DIRECT | GO:0005886~plasma membrane | 190 | 22.43211 | 0.002588 | 0.645373 | 0.071374 |
| GOTERM_CC_DIRECT | GO:0071944~cell periphery | 7 | 0.826446 | 0.003403 | 0.744197 | 0.086882 |
| GOTERM_CC_DIRECT | GO:0005887~integral component of plasma membrane | 75 | 8.854782 | 0.00353 | 0.756989 | 0.08462 |
| GOTERM_CC_DIRECT | GO:0005871~kinesin complex | 8 | 0.94451 | 0.003877 | 0.788593 | 0.087357 |
| GOTERM_CC_DIRECT | GO:0000811~GINS complex | 3 | 0.354191 | 0.004271 | 0.819495 | 0.090728 |
| GOTERM_CC_DIRECT | GO:0005876~spindle microtubule | 7 | 0.826446 | 0.006267 | 0.919126 | 0.123976 |
| GOTERM_CC_DIRECT | GO:0031262~Ndc80 complex | 3 | 0.354191 | 0.008325 | 0.964705 | 0.15397 |
| GOTERM_CC_DIRECT | GO:0043025~neuronal cell body | 22 | 2.597403 | 0.010018 | 0.982176 | 0.174506 |
| GOTERM_CC_DIRECT | GO:0030496~midbody | 12 | 1.416765 | 0.010643 | 0.986158 | 0.176794 |
| GOTERM_CC_DIRECT | GO:0048471~perinuclear region of cytoplasm | 35 | 4.132231 | 0.022856 | 0.999904 | 0.331097 |
| GOTERM_CC_DIRECT | GO:0030424~axon | 16 | 1.88902 | 0.024078 | 0.999942 | 0.333828 |
| GOTERM_CC_DIRECT | GO:0005783~endoplasmic reticulum | 44 | 5.194805 | 0.026391 | 0.999977 | 0.348143 |
| GOTERM_CC_DIRECT | GO:0031298~replication fork protection complex | 3 | 0.354191 | 0.026992 | 0.999982 | 0.34359 |
| GOTERM_CC_DIRECT | GO:0005796~Golgi lumen | 9 | 1.062574 | 0.030651 | 0.999996 | 0.369468 |
| GOTERM_CC_DIRECT | GO:0030425~dendrite | 21 | 2.479339 | 0.033741 | 0.999999 | 0.387578 |
| GOTERM_CC_DIRECT | GO:0005881~cytoplasmic microtubule | 6 | 0.708383 | 0.044642 | 1 | 0.467362 |

Table S4: The results of Kyoto Encyclopedia of Genes and Genomes analysis in the female subject

| **Category** | **Term** | **Count** | **%** | **P-Value** | **Bonferroni** | **Benjamini** |
| --- | --- | --- | --- | --- | --- | --- |
| KEGG_PATHWAY | hsa04512: ECM-receptor interaction | 17 | 2.007084 | 1.23E-07 | 2.96E-05 | 2.96E-05 |
| KEGG_PATHWAY | hsa04514: Cell adhesion molecules (CAMs) | 17 | 2.007084 | 8.61E-05 | 0.020544 | 0.010326 |
| KEGG_PATHWAY | hsa04060: Cytokine-cytokine receptor interaction | 19 | 2.243211 | 0.004895 | 0.693482 | 0.325753 |
| KEGG_PATHWAY | hsa04510: Focal adhesion | 17 | 2.007084 | 0.004979 | 0.699712 | 0.259739 |
| KEGG_PATHWAY | hsa04974: Protein digestion and absorption | 10 | 1.180638 | 0.005905 | 0.760038 | 0.248328 |
| KEGG_PATHWAY | hsa04978: Mineral absorption | 7 | 0.826446 | 0.005917 | 0.760721 | 0.212076 |
| KEGG_PATHWAY | hsa05146: Amoebiasis | 11 | 1.298701 | 0.006716 | 0.802878 | 0.207046 |
| KEGG_PATHWAY | hsa05144: Malaria | 7 | 0.826446 | 0.010015 | 0.911584 | 0.261558 |
| KEGG_PATHWAY | hsa04062: Chemokine signaling pathway | 15 | 1.770956 | 0.01091 | 0.928907 | 0.254539 |
| KEGG_PATHWAY | hsa05322: Systemic lupus erythematosus | 11 | 1.298701 | 0.03068 | 0.999452 | 0.528095 |
| KEGG_PATHWAY | hsa00670: One carbon pool by folate | 4 | 0.472255 | 0.038186 | 0.999916 | 0.573869 |
| KEGG_PATHWAY | hsa04115: p53 signaling pathway | 7 | 0.826446 | 0.040791 | 0.999956 | 0.56673 |

Table S5:　The results of the male subject via gene ontology analysis

| **Category** | **Term** | **Count** | **%** | **P-Value** | **Bonferroni** | **Benjamini** |
| --- | --- | --- | --- | --- | --- | --- |
| GOTERM_CC_DIRECT | GO:0005833~hemoglobin complex | 6 | 0.96 | 5.22E-06 | 0.001513 | 0.001513 |
| GOTERM_CC_DIRECT | GO:0016021~integral component of membrane | 162 | 25.92 | 4.46E-05 | 0.012839 | 0.00644 |
| GOTERM_CC_DIRECT | GO:0005886~plasma membrane | 131 | 20.96 | 2.26E-04 | 0.063534 | 0.021643 |
| GOTERM_CC_DIRECT | GO:0005615~extracellular space | 51 | 8.16 | 0.001221 | 0.298311 | 0.084758 |
| GOTERM_CC_DIRECT | GO:0031430~M band | 5 | 0.8 | 0.001978 | 0.436796 | 0.108476 |
| GOTERM_CC_DIRECT | GO:0005759~mitochondrial matrix | 18 | 2.88 | 0.002338 | 0.492735 | 0.106957 |
| GOTERM_CC_DIRECT | GO:0031838~haptoglobin-hemoglobin complex | 3 | 0.48 | 0.003304 | 0.616985 | 0.128115 |
| GOTERM_CC_DIRECT | GO:0005887~integral component of plasma membrane | 51 | 8.16 | 0.003399 | 0.627453 | 0.116111 |
| GOTERM_CC_DIRECT | GO:0016324~apical plasma membrane | 16 | 2.56 | 0.004458 | 0.726273 | 0.134076 |
| GOTERM_CC_DIRECT | GO:0071682~endocytic vesicle lumen | 4 | 0.64 | 0.006001 | 0.825449 | 0.160168 |
| GOTERM_CC_DIRECT | GO:0030018~Z disc | 9 | 1.44 | 0.007375 | 0.883133 | 0.177294 |
| GOTERM_CC_DIRECT | GO:0005739~mitochondrion | 46 | 7.36 | 0.011415 | 0.964183 | 0.242282 |
| GOTERM_CC_DIRECT | GO:0030315~T-tubule | 5 | 0.8 | 0.012453 | 0.973595 | 0.243878 |
| GOTERM_CC_DIRECT | GO:0031225~anchored component of membrane | 8 | 1.28 | 0.0187 | 0.995807 | 0.323636 |
| GOTERM_CC_DIRECT | GO:0014704~intercalated disc | 5 | 0.8 | 0.022081 | 0.998459 | 0.350586 |
| GOTERM_CC_DIRECT | GO:0043005~neuron projection | 12 | 1.92 | 0.027545 | 0.999696 | 0.397252 |
| GOTERM_CC_DIRECT | GO:0016323~basolateral plasma membrane | 10 | 1.6 | 0.029129 | 0.999811 | 0.396067 |
| GOTERM_CC_DIRECT | GO:0072562~blood microparticle | 9 | 1.44 | 0.029647 | 0.999838 | 0.384218 |
| GOTERM_CC_DIRECT | GO:0009986~cell surface | 21 | 3.36 | 0.037168 | 0.999983 | 0.439039 |
| GOTERM_CC_DIRECT | GO:0033017~sarcoplasmic reticulum membrane | 4 | 0.64 | 0.046825 | 0.999999 | 0.501113 |

Table S6:　The results of Kyoto Encyclopedia of Genes and Genomes analysis in the male subject

| **Category** | **Term** | **Count** | **%** | **P-Value** | **Bonferroni** | **Benjamini** |
| --- | --- | --- | --- | --- | --- | --- |
| KEGG_PATHWAY | hsa01100: Metabolic pathways | 45 | 0.074399 | 0.005675 | 0.7058025 | 0.7058025 |
| KEGG_PATHWAY | hsa00280: Valine, leucine and isoleucine degradation | 6 | 0.00992 | 0.006051 | 0.7287909 | 0.4792226 |
| KEGG_PATHWAY | hsa00410: beta-Alanine metabolism | 5 | 0.008267 | 0.00701 | 0.7796379 | 0.3959879 |
| KEGG_PATHWAY | hsa00330: Arginine and proline metabolism | 6 | 0.00992 | 0.007871 | 0.8171384 | 0.346071 |
| KEGG_PATHWAY | hsa04260: Cardiac muscle contraction | 7 | 0.011573 | 0.010853 | 0.9042648 | 0.3745188 |
| KEGG_PATHWAY | hsa00071: Fatty acid degradation | 5 | 0.008267 | 0.020179 | 0.9875092 | 0.5183126 |
| KEGG_PATHWAY | hsa04080: Neuroactive ligand-receptor interaction | 14 | 0.023146 | 0.02108 | 0.9897512 | 0.4802311 |
| KEGG_PATHWAY | hsa01200: Carbon metabolism | 8 | 0.013226 | 0.022746 | 0.9928946 | 0.4611736 |
| KEGG_PATHWAY | hsa00640: Propanoate metabolism | 4 | 0.006613 | 0.031828 | 0.9990455 | 0.5382375 |
| KEGG_PATHWAY | hsa01130: Biosynthesis of antibiotics | 11 | 0.018186 | 0.039433 | 0.9998248 | 0.5789407 |
